# Cytotoxic Effects of Tetracycline Analogues (Doxycycline, Minocycline and COL-3) in Acute Myeloid Leukemia HL-60 Cells

**DOI:** 10.1371/journal.pone.0114457

**Published:** 2014-12-15

**Authors:** Hairong Song, Mona Fares, Kim R. Maguire, Åke Sidén, Zuzana Potácová

**Affiliations:** 1 Experimental Cancer Medicine, Clinical Research Center Novum, Department of Laboratory Medicine, Karolinska University Hospital Huddinge, Karolinska Institute, Stockholm, Sweden; 2 Department of Neurology, Karolinska University Hospital, Stockholm Sweden; Emory University, United States of America

## Abstract

Tetracycline analogues (TCNAs) have been shown to inhibit matrix metalloproteinases and to induce apoptosis in several cancer cell types. In the present study, the cytotoxic effects of TCNAs doxycycline (DOXY), minocycline (MINO) and chemically modified tetracycline-3 (COL-3) were investigated in the human acute myeloid leukemia HL-60 cell line. Cells were incubated with TCNAs in final concentrations of 0.5–100 µg/ml for 24 h. Viability of the leukemic cells was inhibited in a concentration-dependent manner using resazurin assay. The estimated IC_50s_ were 9.2 µg/ml for DOXY, 9.9 µg/ml for MINO and 1.3 µg/ml for COL-3. All three TCNAs induced potent cytotoxic effects and cell death. Apoptosis, which was assessed by morphological changes and annexin V positivity, was concentration- and time-dependent following incubation with any one of the drugs. TCNAs induced DNA double strand breaks soon after treatment commenced as detected by γH2AX and western blot. The loss of mitochondrial membrane potential (Δψm), caspase activation and cleavage of PARP and Bcl-2 were observed; however, the sequence of events differed among the drugs. Pancaspase inhibitor Z-VAD-FMK improved survival of TCNAs-treated cells and decreased TCNAs-induced apoptosis. In summary, we demonstrated that TCNAs had a cytotoxic effect on the HL-60 leukemic cell line. Apoptosis was induced via mitochondria-mediated and caspase-dependent pathways in HL-60 cells by all three TCNAs. COL-3 exerted the strongest anti-proliferative and pro-apoptotic effects in concentrations that have been achieved in human plasma in reported clinical trials. These results indicate that there is a therapeutic potential of TCNAs in leukemia.

## Introduction

Leukemia is a heterogeneous group of hematopoietic malignancies with worldwide distribution. The World Health Organization has developed a consensus-based classification for hematopoietic and lymphoid neoplasms which are defined based on morphological, clinical and biological features [1]. As the eleventh most common cancer, leukemias (all subtypes included) are relatively rare diseases. The National Cancer Institute estimates that in the United States 52 380 individuals will be diagnosed with some form of leukemia in the year 2014, and thus, leukemia represents about 3% of all new cancer cases [2]. Recently, the incidence of 13.0 per 100 000 persons has been reported in the USA and 10.4 per 100 000 persons in the United Kingdom; however, the incidence of different forms of the disease varies with age. Leukemia is the sixth leading cause of cancer associated death in the United States and eleventh in the United Kingdom with rates of 7.1 and 4.9 per 100 000 persons per year respectively [2], [3]. There are four main types of leukemia: acute myeloid leukemia (AML), acute lymphoblastic leukemia (ALL), chronic myeloid leukemia (CML) and chronic lymphocytic leukemia (CLL). AML is a characterized by differentiation arrest in the myeloid lineage resulting in an accumulation of immature progenitors in the bone marrow and, consequently, hematopoietic failure. In adults, the incidence increases from 1.3 per 100 000 in adolescence to over 15 per 100 000 in the seventh and eighth decade of life [4]. Forty years ago the remission-induction treatment for AML consisting of cytarabine and daunorubicin (7+3) was established in its current form. Few therapeutic approaches for malignant diseases have remained essentially unchanged for such a long period [5]. Regimen intensification and improvements in supportive care have resulted in better outcome for younger AML patients. Nevertheless, age remains an independent adverse prognostic factor for the disease. In older patients, the benefits of intensive therapy are often neutralized by increased therapy related mortality. Despite a better understanding of the unique biological and clinical features of AML in older patients, and development of new drugs, the co-morbidities and high susceptibility to treatment-related toxicity still limit treatment success [6]. Therefore, new treatment strategies are needed in order to maximize the therapeutic benefit and minimize treatment-related toxicity in this population. Those strategies may consist of better selection of patients who tolerate and benefit from intensive induction chemotherapy, as well as the development of novel agents, or a combination of both.

Anti-tumor antibiotics, such as actinomycin and anthracyclines, were discovered and developed for antibiotic purposes but proved to be too toxic for the treatment of infectious diseases [7]. However, their cytotoxic potential has been used in the treatment of cancer. There are other antibiotics, such as tetracyclines, that are currently being used in the treatment of infectious diseases and that have also been found to possess anti-neoplastic properties [8].

The tetracyclines were discovered in late 1940’s as broad-spectrum antibiotics. Currently, tetracyclines may be classified as natural products, semisynthetic compounds and chemically modified tetracyclines (CMTs). The bacteriostatic effect of the tetracyclines is mediated by inhibition of protein synthesis by preventing the attachment of the aminoacy1 t-RNA to the ribosome in the bacterial cell [9]. Tetracyclines have also been found to inhibit the activity of matrix metalloproteinases (MMPs) and mitochondrial protein synthesis, and to act as ionophores which bind to divalent metal cations (such as Ca^2+^ and Mg^2+^) forming lipid-soluble complexes that facilitate their transport across hydrophobic membranes [8]. Chemically modified tetracycline-3 (6-demethyl, 6-deoxy, 4-dedimethylamino tetracycline; COL-3) does not have an antibiotic capacity due to the removal of a dimethylamino group from carbon-4 in the A ring [10].

TCNAs have been shown to induce *in vitro* cell death in several neoplastic cell lines (prostate, breast, colorectal and pancreatic cancer, melanoma, osteosarcoma and leukemia) [11]–[14]. Cell death may be classified into several types as defined by morphological and biochemical features [15]. Activation of apoptosis is a key mechanism by which cytotoxic drugs eliminate cancer cells; however, the sequence of events may differ depending on the mechanism of action of the drug and/or the cell type. Apoptosis can occur by mitochondrial (intrinsic) or death receptor (extrinsic) pathways and caspases serve as main effectors in this process [16]. Mitochondria are vital for cellular bioenergetics and play a central role in determining the point-of-no-return of apoptosis [17]. Activation of the mitochondrial pathway results in biochemical changes, such as a loss of mitochondrial membrane potential (Δψm), permeability transition and release of cytochrome c (cyt c) into cytosol. Upon activation, a cyt c/Apaf-1/caspase-9 apoptosome complex is formed which results in activation of caspase-3. In the final process of apoptosis, the genomic DNA is degraded into fragments [15]. The TCNAs-induced apoptosis in cancer cells was reported to be associated with Bcl-2 inhibition, caspase activation and mitochondrial response such as cyt c release and loss of Δψm [12], [14].

In this study, we investigated the cytotoxic effects of TCNAs DOXY, MINO and COL-3 in the human myeloid leukemia cell line HL-60 that was originally established and classified as acute promyelocytic leukemia. However, the cell line was later approved to be acute myeloid leukemia M2 according to the French-American-British classification, and thus, may serve as a model for AML [18]. We report that TCNAs reduced the viability of the HL-60 cells in a concentration-dependent manner. The HL-60 cells underwent mitochondria-mediated and caspase-dependent apoptosis. COL-3 was shown to have the strongest antiproliferative and pro-apoptotic effects. Thus, TCNAs might have therapeutic potential in AML.

## Materials and Methods

### Chemical reagents

Doxycycline hyclate and minocycline hydrochloride were purchased from Sigma Aldrich (Stockholm, Sweden) and COL-3 was generously provided by CollaGenex Pharmaceuticals Inc (Newtown, PA, USA) and Galderma R&D (Sophia-Antipolis, France). Stock solutions of DOXY and MINO were prepared in distilled water (dH_2_O) in a concentration of 5 mg/ml and stocks of COL-3 in dimethyl sulfoxide (DMSO; Sigma Aldrich, Stockholm, Sweden) in a concentration of 50 mg/ml and stored at −20°C.

The other materials were purchased as follows: Z-VAD-FMK (Bachem AG, Bubendorf, Switzerland); etoposide (Bristol-Myers Squibb, Bromma, Sweden); RPMI 1640 medium, Dulbecco’s phosphate-buffer saline (PBS) and fetal bovine serum (FBS) (Invitrogen AB, Stockholm, Sweden); resazurin (R&D System, Stockholm, Sweden); cOmplete mini protease inhibitor cocktail (Roche AB, Stockholm, Sweden); propidium iodide (Sigma Aldrich) and annexin V (BD, Stockholm, Sweden). All other chemicals and solvents were of analytical grade and purchased from Merck, Darmstadt, Germany.

### Cell culture

The myeloid HL-60 cell line was purchased from DSMZ (Braunschweig, Germany). Cells were grown in RPMI 1640 medium supplemented with 10% heat-inactivated FBS (complete medium) at 37°C in 95% humidified 5% CO_2_ atmosphere. HL-60 cells were seeded at a concentration of 2×10^5^ cells/ml. All experiments were performed while cells were in exponential growth.

### Treatment

The stock solutions of TCNAs were diluted in complete medium immediately prior to use. To assess the IC_50_, cells were incubated with DOXY, MINO and COL-3 for 24 h in final concentrations of 0.5, 1, 2.5, 5, 10, 25, 50 and 100 µg/ml. In experiments using general caspase inhibitor, cells were pre-treated with Z-VAD-FMK in a concentration of 100 µM for 1 h, and then incubated with DOXY or MINO in concentrations of 25 µg/ml or COL-3 in concentrations of 2.5 µg/ml or 5 µg/ml for 6 h and 24 h. The concentrations and incubation times for other experiments are stated within the appropriate text. To test for solvent toxicity, cells were incubated with DMSO in a final concentration of 0.2%. Cells treated with etoposide in a final concentration of 6 µg/ml served as positive control for apoptosis. Cells incubated in complete medium were used as a negative control whenever appropriate.

### Assessment of cell viability

Cell viability was determined using resazurin assay. Ten thousands cells were seeded in triplicates on 96 well black microplates and incubated with TCNAs for 24 h. Resazurin was then added in a final concentration of 10% and cells were further incubated for 2 h at 37°C. Fluorescence was read using FLUOstar Optima (BMG Labtech GmbH, Offenburg, Germany) at a wavelength of 590 nm.

### Assessment of apoptosis

Apoptosis was assessed using annexin V/propidium iodide staining and flow cytometry. HL-60 cells were incubated with DOXY or MINO in concentrations of 5, 10, 25 and 50 µg/ml or COL-3 in concentrations of 0.75, 1, and 5 µg/ml until 48 h. Cells were then stained with annexin V and propidium iodide (PI) in concentrations of 125 ng/ml and 5 µg/ml, respectively, and incubated for 15 min at room temperature (RT) in the dark. Samples were analyzed using FACScan flow cytometer and CELL Quest software (BD, San Jose, CA, USA). Apoptotic cells were also identified using morphological criteria, such as condensed chromatin, fragmented nuclei and formation of apoptotic bodies, in May-Grünwald-Giemsa stained cytospun slides.

### Determination of mitochondrial membrane potential (Δψm)

To assess the effect of TCNAs on mitochondria, HL-60 cells were incubated with DOXY or MINO in final concentrations of 25 and 50 µg/ml or COL-3 in final concentrations of 0.5, 1, 2.5 and 5 µg/ml for 2, 4, 6 and 24 h. Δψm was assessed using tetramethylrhodamine methyl ester (TMRM) (Molecular Probes, Carlsbad, CA, USA). Following the incubation with TCNAs, cells were incubated with 25 nM TMRM in PBS at 37°C for 30 min. After washing, samples were analyzed using FACScan flow cytometer and CELL Quest software.

### Western blot analysis

Cells were lysed in lysis buffer (50 mM Tris-NaCl pH 7.4, cOmplete mini protease inhibitor cocktail, 0.1 M PMSF, Triton-X 100) for 30 minutes on ice. For detection of poly (ADP-ribose) polymerase (PARP), the cells were lysed in PARP-extraction buffer (62.5 mM Tris pH 6.8, 6 M urea, 2% SDS, 10% Glycerol and 0.001% bromophenol blue) for 15 min at RT and then for another 5 min at 95°C. Cells lysates were centrifuged at 10 000 g for 10 min at 4°C and the supernatants were stored at −70°C.

Cellular fractionation was performed after lysing the cells in digitonin cytosolic buffer (5 mM Tris-HCl pH 7.4, 5 mM succinic acid pH 6.3, 10 mM MgCl_2_.6H2O, 0.5 mM EDTA, 147.5 mM KCl, 5 mM KH_2_PO4 and 0.005% digitonin) for 30 min on ice. The samples were centrifuged at 10 000 g for 10 min at 4°C. The supernatant (cytosolic fraction) was collected and the pellet was re-suspended in PBS containing cOmplete mini protease inhibitor cocktail (pellet fraction).

Protein concentrations were measured using BCA protein assay system (Pierce, Rockford, IL, USA) according to the manufacturer’s recommendations. Cellular proteins (20 µg) were separated by 12% SDS-PAGE and transferred to a PVDF membrane. The membranes were blocked in 5% non-fat dry milk solution at 4°C overnight and then incubated with primary antibody for 2 h at RT. The following antibodies were used: rabbit primary antibodies against caspase-2, -8 or -9 (BD, Stockholm, Sweden); caspase-3, γH2AX (20E3) (Cell Signaling, Stockholm, Sweden); actin (Sigma, St. Louis, MO, USA) or mouse primary antibodies against caspase-7 and cytochrome c (cyt c) (BD); Bcl-2 (Dako Sweden AB, Stockholm, Sweden); caspase-2 (C2) (Cell Signaling); PARP (Oncogene Research Products, Boston, MA, USA). The dilution of primary antibodies was 1∶5000 except for caspase-3, PARP and actin (1∶1000) and γH2AX (1∶500). After washing, the membranes were incubated with peroxidase-conjugated (Amersham Pharmacia Biotech AB, Uppsala Sweden), or IRDye 800CW or IRDye 680CW conjugated (LI-COR, Lincoln, Nebraska, USA) secondary anti-rabbit or anti-mouse antibodies in dilution 1∶10 000 except for caspase-2 (C2), caspase-3, PARP and actin (1∶2000) for 1 h at room temperature. The proteins were visualized using SuperSignal West Pico Chemiluminescent Substrate (Pierce, Rockford, IL, USA) or ODYSSEY imaging system (LI-COR). Quantitative analysis of specific bands was performed by densitometry analysis using Image J software. Since actin remained intact after incubation with any of the drugs, actin was further used as a marker of equal protein loading.

### Data analysis

Viability of treated cells obtained from resazurin assay was expressed as a percentage of the viability of untreated cells. The 50% inhibitory concentrations (IC_50s_) were determined as the concentrations of the TCNAs causing 50% decrease in cell viability. The data were fitted into a nonlinear regression model for normalized response with variable slope using GraphPad Prism 6 (GraphPad Software, Inc. La Jolla, CA, USA).

## Results

### Effect of TCNAs on cell viability

**Figure 1:**
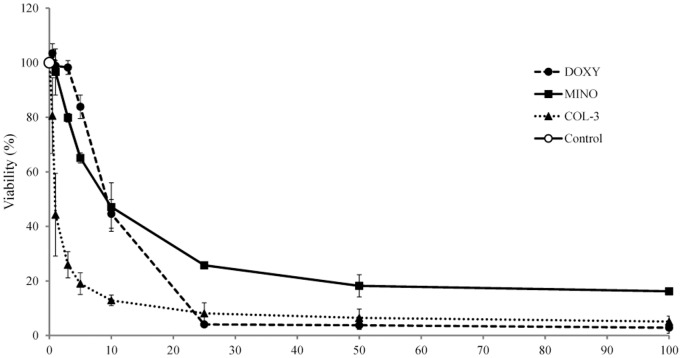
The effect of TCNAs on the viability of human leukemic HL-60 cell line. HL-60 cells were incubated with DOXY, MINO or COL-3 in concentrations within the range 0.5–100 µg/ml for 24 h. Cells incubated in DMSO in a final concentration of 0.2% were used as controls for solvent toxicity and cells incubated in complete medium served as controls. Viability was studied using resazurin fluorescence assay and expressed as percent of the control. Results are presented as a mean ± SD of three independent experiments.

Incubation of the human myeloid HL-60 cell line with all three studied TCNAs for 24 h reduced the cell viability in a concentration-dependent manner. The IC_50_ for DOXY was 9.2 (95% CI 8.79–9.66; R^2^ 0.995), for MINO were 9.9 (95% CI 8.61–11.29; R^2^ 0.974) and for COL-3 1.3 (95% CI 1.09–1.56; R^2^ 0.922) (Fig. 1). DMSO in concentration 0.2% did not affect the cell viability. Thus, COL-3 was shown to be the most effective in HL-60 cells.

### TCNAs-induced apoptosis

**Figure 2:**
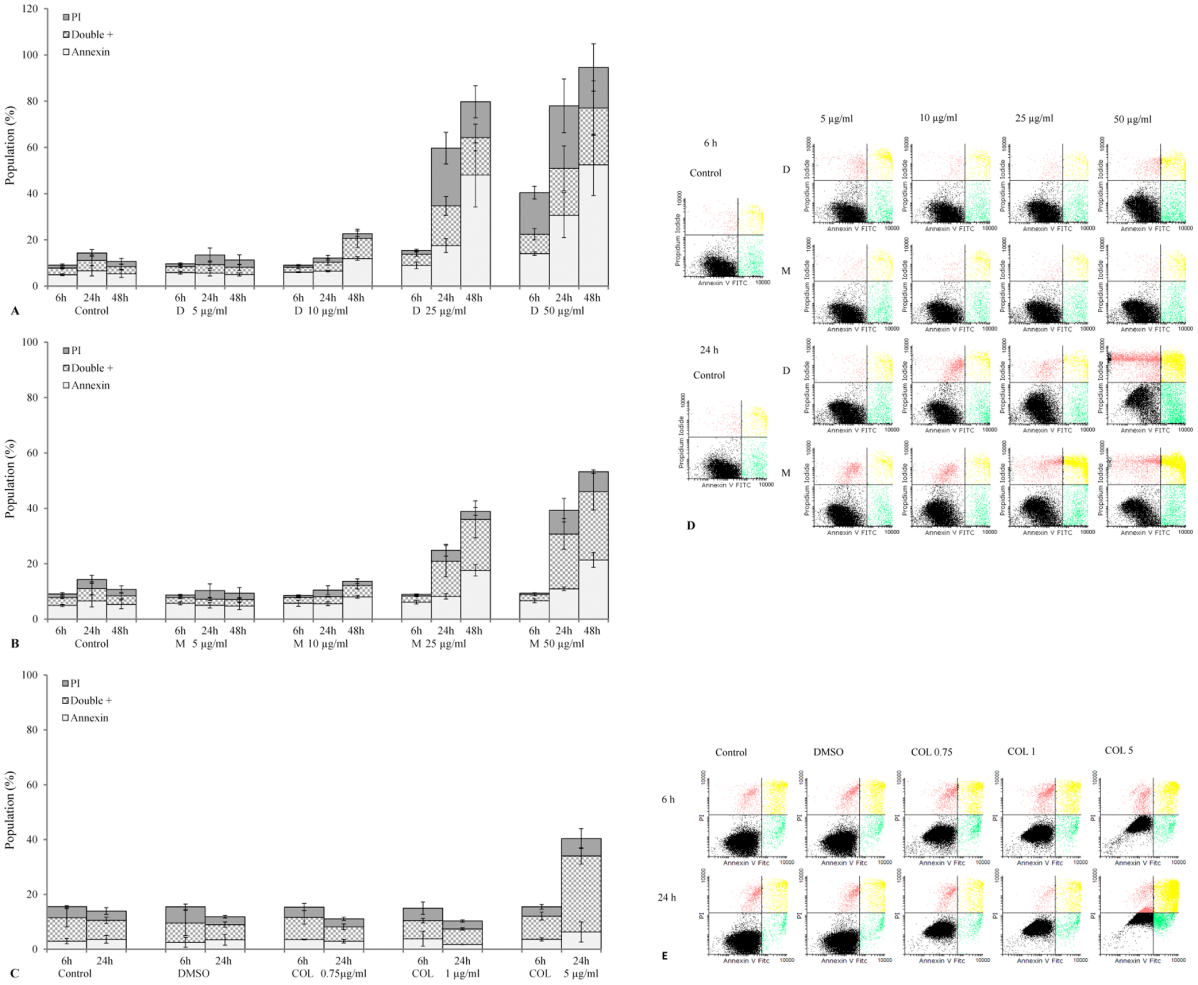
TCNAs-induced apoptosis. HL-60 cells were incubated with DOXY (A) and MINO (B) in concentrations of 5, 10, 25 and 50 µg/ml or COL-3 (C) in concentrations of 0.75, 1, and 5 µg/ml until 48 h. Cells incubated in DMSO in a final concentration of 0.2% were used as controls for solvent toxicity and cells incubated in complete medium served as controls. Cells were stained with annexin V and PI for 15 min at room temperature in the dark. Twenty thousand events were acquired and analyzed using flow cytometry. Subpopulations are expressed as percentages of total events. Results are expressed as a mean ± SD of three independent experiments. (D), (E): Examples on flow cytometric analysis of TCNAs-induced apoptosis using annexin V/PI assay. D: DOXY, M: MINO COL: COL-3 and PI: propidium iodide.

DOXY, MINO and COL-3 induced apoptosis in HL-60 cells as detected by positive staining for annexin V. Annexin V positive cells and annexin V/PI double positive cells were observed in a concentration- and time-dependent pattern. Annexin V+ cells were observed following 6 h incubation with DOXY, and levels increased over the incubation period. The double positive cells increased followed by appearance of PI+ cells (Fig. 2A and 2D). In cells treated with MINO, the annexin V and/or annexin V/PI double positive cells followed a similar pattern; however, they were initially detected at 24 h (Fig. 2B and 2D). In COL-3, the double positive cells were detected at 24 h (Fig. 2C and 2E) and only a minor increase of annexin V+ cells was observed. Nevertheless, apoptotic morphology of treated cells was confirmed in May-Grünwald-Giemsa staining on cytospun slides in all treatments.

### Effect of TCNAs on Δψm, Bcl-2 and cytochrome c

**Figure 3:**
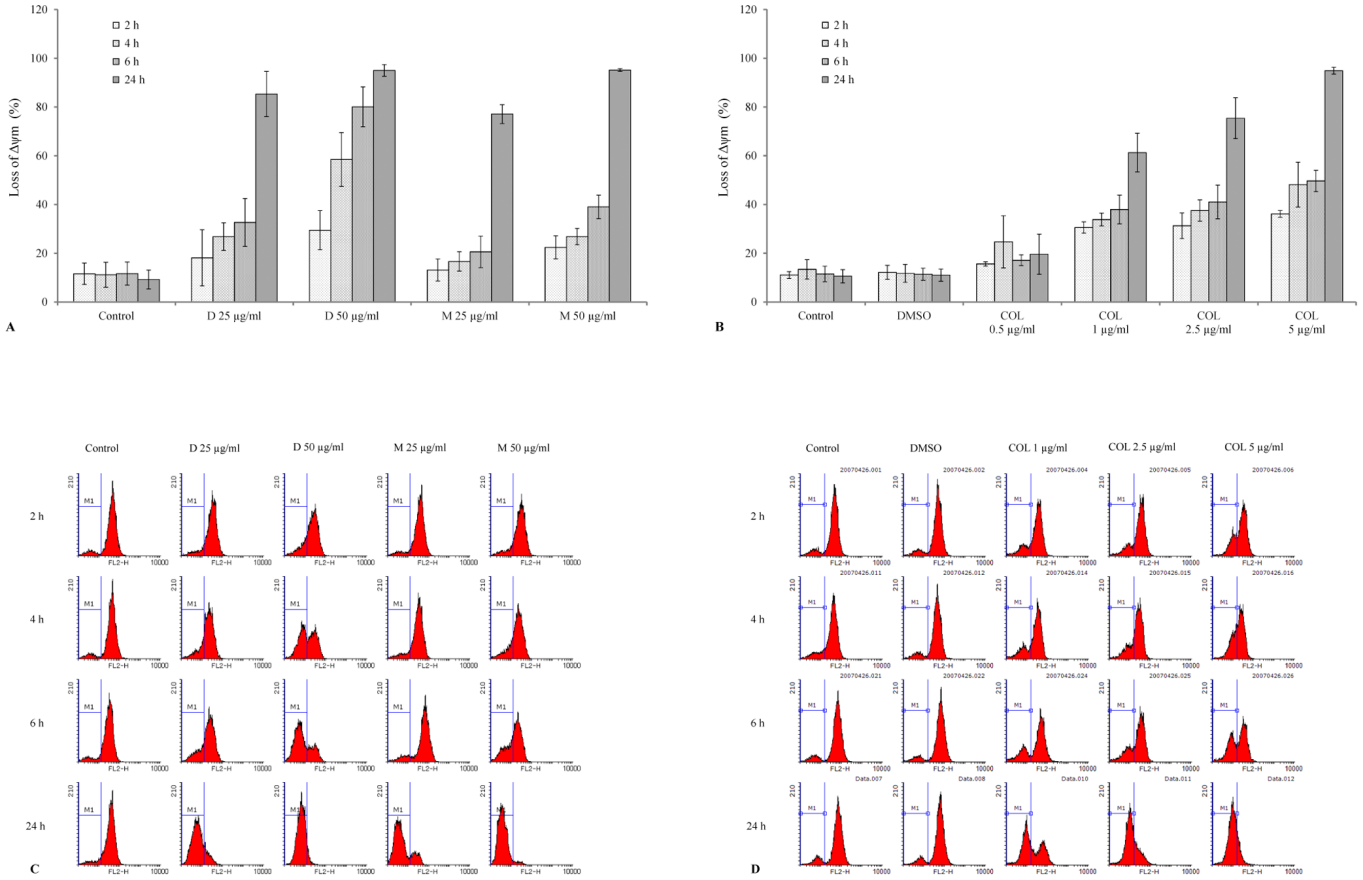
The loss of mitochondrial membrane potential (Δψm) in TCNAs-induced apoptosis. Mitochondrial membrane potential was assessed using tetramethylrhodamine methyl ester (TMRM) and flow cytometry. (A) HL-60 cells were incubated with DOXY or MINO in final concentrations of 25 and 50 µg/ml or (B) COL-3 in final concentrations of 0.5, 1, 2.5 and 5 µg/ml for 2, 4, 6 and 24 h. Cells incubated in DMSO in a final concentration of 0.2% were used as controls for solvent toxicity and cells incubated in complete medium served as controls. Results are expressed as mean ± SD of three independent experiments. (C), (D): Example on flow cytometric analysis of loss of Δψm using TMRM assay. D: DOXY, M: MINO, COL: COL-3.

**Figure 4:**
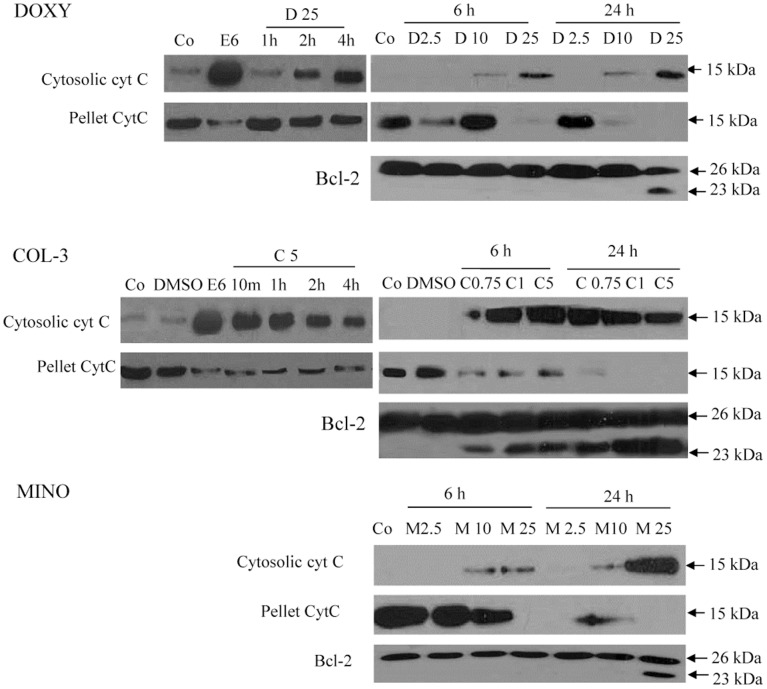
The role of mitochondria in TCNAs-induced apoptosis. Cytosolic translocation of cytochrome c (cyt c) and Bcl-2 cleavage were assessed with Western blot. Cells were incubated with DOXY or MINO in final concentrations of 2.5, 10 and 25 µg/ml or COL-3 in final concentrations of 0.75, 1 and 5 µg/ml until 24 h. DMSO (0.2%) treated cells served as controls for solvent toxicity, etoposide (6 µg/ml) treated cells as positive controls for apoptosis and cells incubated in complete medium as controls. Co: control, D: DOXY, M: MINO, C: COL-3 and E: etoposide.

Loss of Δψm was observed as early as at 2 h of incubation with COL-3 in all tested concentrations (Fig. 3B and 3D). Also DOXY and MINO induced loss of Δψm at 2 h post incubation start, but only in the highest tested concentration (50 µg/ml) (Fig. 3A and 3C). The decrease in Δψm progressed in a concentration- and time-dependent manner with almost complete loss of Δψm observed at 24 h post incubation with COL-3 in concentration of 5 µg/ml and DOXY and MINO in concentrations of 50 µg/ml, respectively (Fig. 3A–3D). DMSO did not affect Δψm compared to controls incubated with complete medium. Cyt c cytosolic translocation was detected following 10 min and 1 h of incubation with COL-3 and DOXY, respectively, while in cells treated with MINO the cyt c release was observed at 6 h post incubation start. The 23 kDa Bcl-2 fragment was detected following 6 h of incubation with COL-3 and 24 h of incubation with DOXY and MINO (Fig. 4).

### The effect of TCNAs on DNA and PARP

**Figure 5:**
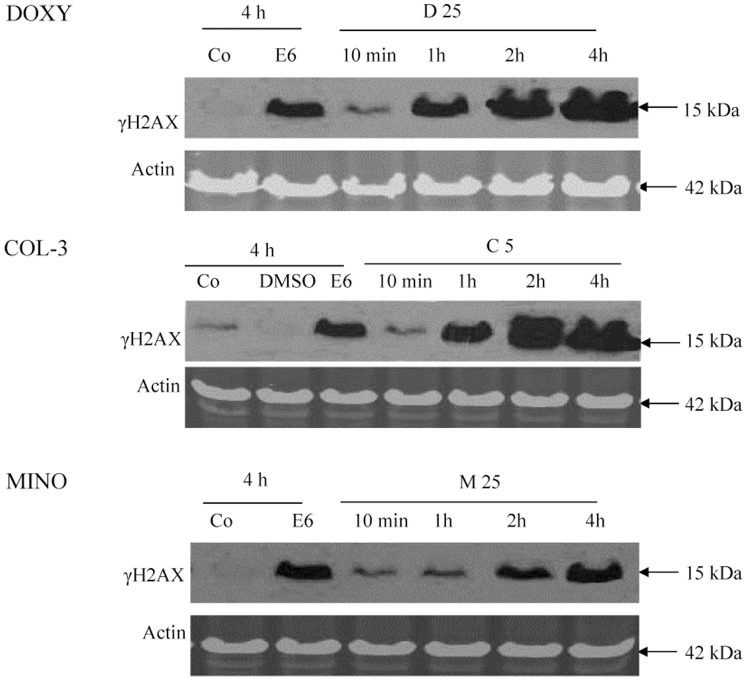
The effect of TCNAs on DNA. HL-60 cells were incubated with DOXY or MINO in final concentrations of 25 µg/ml or COL-3 in a final concentration of 5 µg/ml for 10 min and 1, 2 and 4 h. Cells incubated in DMSO in a final concentration of 0.2% were used as controls for solvent toxicity and cells incubated in complete medium served as controls. Cells incubated with etoposide in a final concentration of 6 µg/ml served as positive controls for apoptosis. The TCNAs-induced DNA damage was assessed using SDS-PAGE and immunostaining for the DNA double strand breaks marker γH2AX. Co: control, E: etoposide, D: DOXY, M: MINO and C: COL-3.

**Figure 6:**
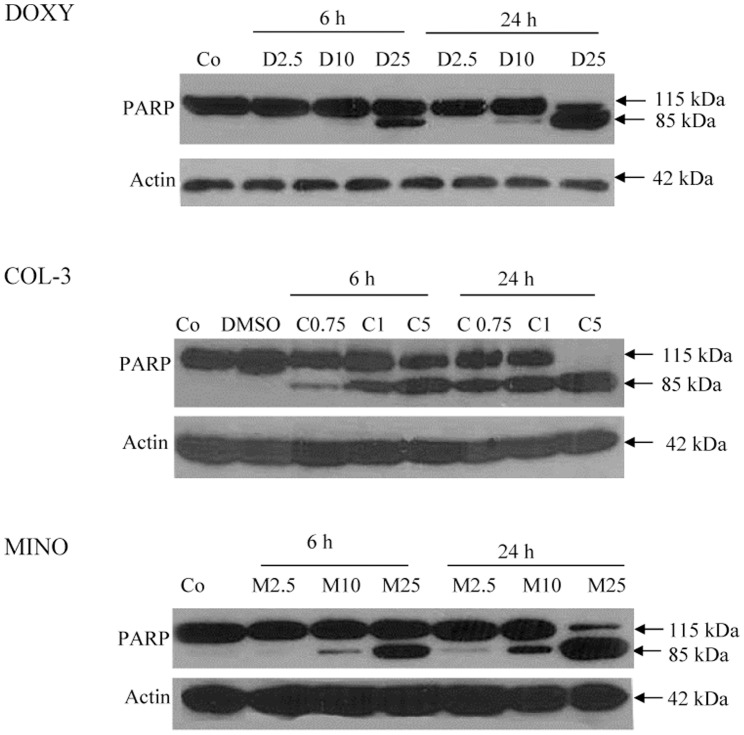
TCNAs-mediated poly (ADP-ribose) polymerase-1(PARP-1) cleavage. HL-60 cells were treated with DOXY or MINO in final concentrations of 2.5, 10 and 25 µg/ml or COL-3 in final concentrations of 0.75, 1 and 5 µg/ml for 6 and 24 h. Cells incubated with DMSO in a final concentration of 0.2% served as controls for solvent toxicity, cells incubated with complete medium served as controls. The pattern of PARP-1 cleavage was assessed using WB. Actin was used as a control for equal protein loading. Co: control, D: DOXY, M: MINO and C: COL-3.

HL-60 cells were incubated with DOXY or MINO in final concentrations of 25 µg/ml or COL-3 in a final concentration of 5 µg/ml for 10 min and 1, 2 and 4 h. TCNAs induced DNA double strand breaks (DSB) as detected by 15 kDa band of γH2AX (Fig. 5). The DNA damage was detected at 1 h of treatment with DOXY and COL-3, and at 2 h with MINO. Cleavage of PARP to the 85 kDa apoptotic fragment was observed in all drugs (Fig. 6).

### Caspase activation in TCNAs-induced apoptosis

HL-60 cells were incubated with DOXY or MINO in concentrations of 2.5, 10 and 25 µg/ml or COL-3 in concentrations of 0.75, 1.0 and 5.0 µg/ml for 2, 4, 6 and 24 h. Activation of caspase-2, -3, -7, -8 and -9 was observed following treatment with all three drugs. However, the pattern of caspase cleavage and detected fragments differed among them (Table 1). The 37 and 33 kDa fragments of caspase-9 were detected at 2 h with DOXY and 6 h with MINO. In COL-3 treated cells the 37 kDa fragment was detected at 2 h, while the 33 kDa fragment was observed at 4 h post incubation start. The 17 and 14 kDa fragments of caspase-3 were observed at 4 h post incubation start with DOXY and COL-3, while at 6 h the 14 kDa was more abundant than the 17 kDa with MINO. Moreover, in cells treated with DOXY and COL-3, a 25 kDa intermediate fragment was detected, but was not observed with MINO treatment. The 32 and 20 kDa fragments of caspase-7 were detected at 6 h post incubation start with DOXY and MINO; however, only a 20 kDa fragment was observed following COL-3 treatment. The long (48 kDa) and short (35 kDa) forms of caspase-2 and 25 kDa fragment were detected following treatment with all three drugs. No 15 kDa fragment was induced by any of the studied drugs. Using another caspase-2 antibody, the 12 kDa fragment was detected at 4 h of incubation with COL-3 only.

**Table 1:** TCNAs-mediated caspase activation.

		Doxycycline 25 µg/ml			
	Fragment	2 h	4 h	6 h	24 h
Caspase-9	37 kDa	+	+	++	+
	33 kDa	+	+	+++	+++
Caspase-2	35 kDa	ND	ND	−	−
	25 kDa	ND	ND	+	++++
	12 kDa	−	−	ND	ND
Caspase-8	23 kDa	−	−	−	+
Caspase-7	32 kDa	ND	ND	+	+++
	20 kDa	ND	ND	++	++++
Caspase-3	17 kDa	−	+++	++	+
	14 kDa	−	++	+++	++++

		**Minocycline 25 µg/ml**			
	**Fragment**	**2 h**	**4 h**	**6 h**	**24 h**
Caspase-9	37 kDa	−	−	++	+
	33 kDa	−	−	+++	+
Caspase-2	35 kDa	ND	ND	−	−
	25 kDa	ND	ND	−	+++
	12 kDa	−	−	ND	ND
Caspase-8	23 kDa	−	−	−	++
Caspase-7	32 kDa	ND	ND	++++	++++
	20 kDa	ND	ND	++++	++++
Caspase-3	17 kDa	−	−	+	−
	14 kDa	−	−	+++	++++

		**COL-3 5 µg/ml**			
	**Fragment**	**2 h**	**4 h**	**6 h**	**24 h**
Caspase-9	37 kDa	++	+++	+++	+++
	33 kDa	+	++	−	−
Caspase-2	35 kDa	ND	ND	−	−
	25 kDa	ND	ND	+	+++
	12 kDa	−	++	ND	ND
Caspase-8	23 kDa	−	−	+	++
Caspase-7	20 kDa	ND	ND	+++	++++
Caspase-3	17 kDa	−	++	+++	++
	14 kDa	−	+	+	+++

HL-60 cells were incubated with DOXY or MINO in final concentrations of 2.5, 10 and 25 µg/ml or COL-3 in final concentrations of 0.75, 1 and 5 µg/ml for 2, 4, 6 and 24 h. Cells incubated in DMSO in a final concentration of 0.2% were used as controls for solvent toxicity and cells incubated in complete medium served as controls. Cells incubated with etoposide in a final concentration of 6 µg/ml served as positive controls for apoptosis. The protein band densities were estimated using the Image J software and each band density in proportion to the control was calculated. The presence of cleaved bands was used as an indicator of caspase activation. The fold changes in proportion to the controls were arranged in the following score to indicate the degree of activation: 5–15 (+), 16–30 (++) 31–60 (+++) and over 60 (++++). ND: not done.

### Effect of pancaspase inhibitor Z-VAD-FMK on TCNAs-induced cytotoxicity

**Figure 7:**
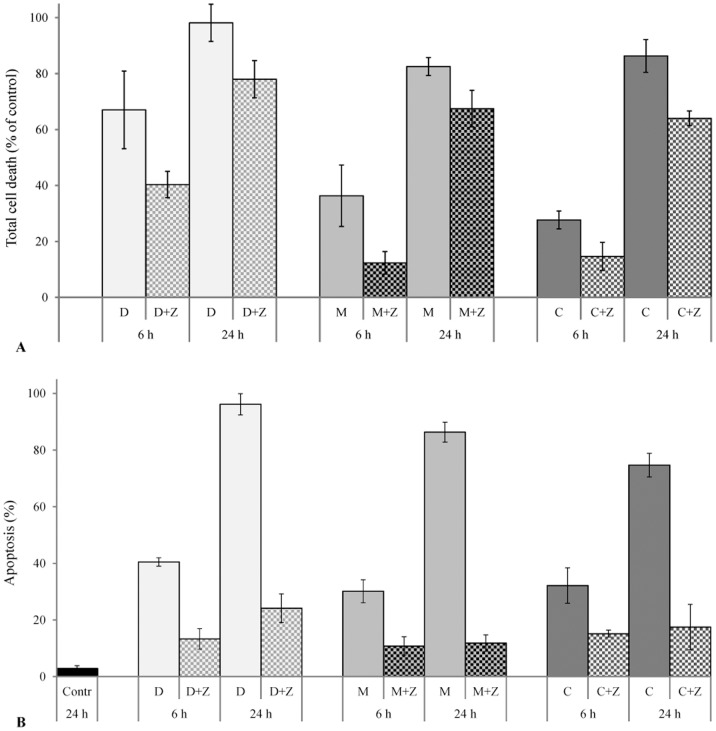
The effect of Z-VAD-FMK on TCNAs-induced cytotoxicity. HL-60 cells were treated with Z-VAD-FMK in a final concentration of 100 µM for 1 h before treatment with TCNAs for 6 h and 24 h. Cells incubated with DMSO in a final concentration of 0.2% served as controls for solvent toxicity, cells incubated in complete medium served as controls. (A) Total cell death was assessed using resazurin viability assay and calculated as 100% - viability %. (B) Apoptosis was assessed by morphological criteria in Giemsa staining. DMSO and Z-VAD-FMK exerted no cytotoxic effects on HL-60 cells compared to controls incubated with complete medium (data not shown on the graph). Results are presented as mean ± SD of three independent experiments. D: DOXY 25 µg/ml, M: MINO 25 µg/ml, Z: Z-VAD-FMK 100 µM; C: COL-3 5 µg/ml in total cell death experiments (A) and 2.5 µg/ml apoptosis experiments (B).

Pre-treatment with the pancaspase inhibitor Z-VAD-FMK resulted in improved viability of TCNAs-treated cells (Fig. 7A). Pre-treatment with Z-VAD-FMK also reduced TCNA-induced apoptosis at 24 h of incubation by 76% in DOXY, 69% in MINO, and 50% in COL-3 (Fig. 7B). No cytotoxic effect of DMSO or Z-VAD-FMK was observed.

## Discussion

Acute myeloid leukemia (AML) is a clonal hematologic disorder that is characterized by differentiation arrest and accumulation of immature myeloid progenitors. Acute myeloid leukemia is the most common leukemia in adults with the peak incidence around the age of 80 years [19]. Tetracyclines have been used for the treatment of different infectious, inflammatory and autoimmune diseases since their introduction into clinics [8], [20]. The expansion of their clinical use is mainly due to the fact that they are well tolerated and safe to use [21]. Furthermore, several studies in cancer cell lines have approved the anticancer effects of TCNAs either as inhibitors of MMPs [14], [22]–[24] or as inducers of apoptosis [13], [25], [26]. Chemically modified tetracyclines possess properties that differ depending on the modification of the tetracycline molecule. COL-3 (6-demethyl, 6-deoxy, 4-dedimethylamino tetracycline) is devoid of the dimethylamino group at the C4 position. This modification does not affect the basic structure of the molecule, but enhances the non-antibiotic properties of COL-3 and improves the antiproliferative and antimetalloproteinase effects [10]. The DOXY (5-hydroxy-alpha-6-deoxytetracycline) molecule contains a hydroxyl group at C5 which may potentiate the antiproliferative effect of the molecule as suggested previously [27]. The MINO (7-dimethylamino-6-demethyl-6-deoxytetracycline) chemical structure contains another dimethylamino group at the C7 on the D ring. This modification is associated with increased antibiotic activity and lower antiproliferative effect compared to DOXY [10].

In this study, we investigated the effect of three TCNAs on the human acute leukemic cell line HL-60. This cell line is established from patients with acute myeloid leukemia M2 according to the FAB classification and is characterized by expression of Bcl-2 and absence of p53 [18], [28]. The HL-60 cells were reported to be sensitive to several conventional anticancer drugs such as chlorambucil and aminopterin [29].

In our study, TCNAs induced a cytotoxic effect in the HL-60 cells in a concentration- and time-dependent manner. The IC_50_ was assessed after 24 h of incubation with TCNAs. The cells were more sensitive to COL-3 with an IC_50_ of 1.3 µg/ml, than to DOXY and MINO with an IC_50_ of 9.2 and 9.9 µg/ml, respectively. These IC_50s_ are in the low range of sensitivities reported in cancer cell lines. IC_50_ within the range 3–10 µg/ml was reported for COL-3, and 10–20 µg/ml for DOXY [30], [31]. MINO has been reported to inhibit proliferation of epithelial cancer cell lines with IC_50_ levels of about 60 µmol/L that correspond to 27 µg/ml [32]. Inhibitory effect of DOXY and COL-3 in HL-60 cells was reported by Tolemeo *et al* [27]. They reported the IC_50s_ for DOXY and COL-3 to be 8 µg/ml and 7 µg/ml, respectively. Our results for DOXY are in agreement with their study, but the IC_50_ for COL-3 is lower in our study. This difference in IC_50_ can be explained by different assays and incubation times used in the studies. We assessed the viability with resazurin assay, and thus examined the effect of TCNAs on the metabolic activity of cell mass, while Tolemeo *et al* assessed the viability of individual cells using trypan blue exclusion assay. They also reported AC_50_ (concentration able to induce 50% apoptosis) for DOXY and COL-3 to be 15 and 16 µg/ml, respectively, but they did not assessed the underlying apoptotic mechanisms. In our study, COL-3 in a concentration of 5 µg/ml induced cell death in HL-60 cells. Other published studies have showed profound apoptotic and growth-inhibitory effects of COL-3 in a concentration of 10 µg/ml in a broad range of human tumor cell lines and xenografts [22], [33], [34].

TCNAs have been reported to induce apoptosis in various cancer cells; however, the reported biochemical events underlying TCNAs-induced cell death differ [11]–[14]. The mitochondrion generates energy for a cell and also controls cell death by releasing death promoting factors into the cytosol. The mitochondrial engagement is centered on two processes. The inner membrane is promoted by the permeability transition pore (PTP), while Bid, BAX and BAK play active roles in outer membrane permeabilization [35]. In our study, COL-3 in a concentration of 5 µg/ml induced cyt c release as early as 10 min and loss of Δψm at 2 h post incubation. Bcl-2 cleavage was detected at 6 h with all tested concentrations (Fig. 4). These results indicate that COL-3 interacted with the mitochondrial PTP on the inner membrane; thereby inducing the collapse of Δψm. Putative pores on the outer mitochondrial membrane were formatted after cleavage of Bcl-2. Furthermore, the DNA damage response to COL-3 was not seen before 1 h of incubation (Fig. 5), which indicates that mitochondria is an early and primary target of COL-3 in HL-60 cells. This observation is supported by the early and strong activation of caspase-9 with COL-3 treatment (Table 1). Similarly, we observed that COL-3 induced a rapid effect on mitochondrion in the chronic myeloid leukemia K562 cell line (unpublished data/manuscript in preparation). Moreover, the DNA damage effect of COL-3 might be secondary to the release of mitochondrial mediators such as apoptosis inducing factor (AIF), as reported by others [14], [36].

DOXY 25 µg/ml induced cyt c release, loss of Δψm and initiated caspase-9 activation at 2 h, while the double strand breaks were detected using γH2AX at 1 h post incubation. These findings indicate that the nucleus may be the primary target for DOXY [37]. Another explanation might be the presence of cytoplasmic stressors such as increased reactive oxygen species (ROS) that can induce DNA damage and mitochondrial response independent of Bcl-2 inhibition [25], [38]. Similarly, after incubation with MINO in a concentration of 25 µg/ml, the DNA damage preceded the mitochondrial response (cyt c release and loss of Δψm) without early cleavage of Bcl-2 protein. However, the response to treatment with MINO was delayed when compared to treatment with DOXY or COL-3 (Fig. 4).

The nucleus plays a central role in cell survival and death. Cellular stressors that induce DNA damage also activate DNA repair mechanisms and can mediate apoptosis only if the damage is extensive. HL-60 cells constitutively express the retinoblastoma protein (Rb) [39] which is an important contributor in cellular response to DNA damage [40]. If the DNA damage is extensive, Rb can activate apoptosis through the mitochondria pathway [41].

The caspase cascade was activated by all three drugs; however, differences in the activation pattern, i.e. sequence of events and/or detected fragments, were observed. We have found an intermediate fragment of caspase-3 in DOXY- and COL-3-induced apoptosis, but this fragment was not observed in MINO-induced apoptosis. A caspase-7 fragment of 32 kDa was observed in treatment with DOXY and MINO, but not in COL-3 treated cells. Furthermore, the long and short form of caspase-2 and fragment of 25 kDa were found in all three treatments, but a 15 kDa fragment was not detected. The origin of the 25 kDa fragment and its role in apoptosis remains unclear. Using another antibody against caspase-2, we were able to detect the 12 kDa fragment in cells treated with COL-3. DOXY and COL-3-induced apoptosis was reported to be associated with activation of caspase-3, -9 and -7 [25], [42]. However, the role of MINO in cell death and cell protection remains controversial. MINO was reported to have a neuroprotective effect mediated through inhibition of inflammation, reduction of cyt c release and inhibition of caspase-9 and -3 activation [43]. However, activation of caspase-3, cleavage of PARP and DNA laddering were reported in ovarian cancer cell lines [32]. In this study, MINO induced cell death was associated with caspase activation, in particular the effector caspase-7. The cytotoxic effect of MINO was also seen to be mediated by mitochondria. The apoptotic effects of all three TCNAs were reduced after preincubation with pancaspase inhibitor Z-VAD-FMK (Fig. 7), confirming that apoptosis induced by TCNAs was caspase-dependent in accordance with what was reported in studies with both DOXY and COL-3 in a colon cancer cell line [14].

The plasma concentrations of COL-3 were studied in humans in Phase I clinical trials. COL-3 was administered orally in doses ranging from 36 to 98 mg/m^2^/day. The plasma steady state concentrations varied from 8.24±3.78 to 10.95±1.34 µg/ml [44]. These concentrations are fivefold higher than the IC_50_ of COL-3 in our study. In Phase II clinical trials, COL-3 showed efficacy and was well tolerated when administered at 50 mg/day [45].

We found that TCNA-induced apoptosis in the AML HL-60 cell line is mitochondria-mediated and caspase-dependent. However, the course of events differed among the tested drugs. COL-3 exerted stronger cytotoxic and pro-apoptotic effects compared to DOXY and MINO. COL-3, in clinically achievable concentrations, had the strongest anti-proliferative and pro-apoptotic effect among tested TCNAs. Thus, TCNAs may have potential in the treatment of AML.
